# Factors Affecting Masticatory Performance of Older Adults Are Sex-Dependent: A Cross-Sectional Study

**DOI:** 10.3390/ijerph192315742

**Published:** 2022-11-26

**Authors:** Hee-Eun Kim, Janet Wallace, Woosung Sohn

**Affiliations:** 1 Department of Dental Hygiene, Gachon University College of Health Science, Incheon 21936, Republic of Korea; 2 Faculty of Medicine and Health, The University of Sydney School of Dentistry, Sydney, NSW 2010, Australia

**Keywords:** functional tooth units, masticatory performance, mixing ability index, older adults, skeletal muscle mass index

## Abstract

This cross-sectional study assessed the oral and physical factors contributing to improvement of the masticatory performance of community-dwelling older adults in South Korea. We enrolled 84 healthy older adults (38 men, 46 women; age, 71.40 ± 5.15 years) and assessed their skeletal muscle mass index (SMI), functional tooth units (FTUs), and mixing ability index (MAI). Associations between variables were analyzed using Spearman’s correlation coefficient, and the effects of SMI and FTUs on the MAI were evaluated through linear multiple regression. FTUs were positively associated with the MAI in men and women (r = 0.339, *p* = 0.038 and r = 0.461, *p* = 0.001, respectively). SMI and FTUs were moderately associated in men (r = 0.459, *p* = 0.004). MAI showed an approximately 4.4 times increase for each FTU in men (B = 4.442, *p* = 0.037); however, after the SMI was added, this effect was no longer significant. In women, the MAI increased by about 6.7 times with each FTU (B = 6.685, *p* = 0.004). FTUs had a significant effect on the MAI only in women with low muscle mass. While there was no significant effect of the SMI on the MAI, its influence should not be overlooked.

## 1. Introduction

With the advancement in research and technology worldwide, subsequent shifts in demographics have drawn the attention of the medical community to the health problems of the older population [1,2]. Public health objectives for the elderly emphasize on “healthy active aging” and “compression of morbidity [3]”; among the multiple approaches to achieve these objectives, optimal nutritional intake is the most important [4]. It has been extensively reported that older people with poor chewing ability are unable to satisfy their nutritional demands. Felton [5] reported a greater risk of malnutrition among edentulous individuals than among dentate or partially dentate individuals. Similarly, Rémond et al. [6] found that postprandial whole-body protein synthesis was about 1.6 percentage points lower among denture wearers than that in dentate individuals. Zhu and Hollis [7] also reported decreased intake of protein and most micronutrients and increased intake of carbohydrates in older individuals with less than 21 teeth. These results suggest that problems with mastication in older individuals are associated with imbalanced nutrient intake. Therefore, management of masticatory function should be prioritized to ensure healthy longevity in the ageing population [8].

However, appropriate maintenance of the masticatory function is not easy because several factors work in combination. Masticatory performance, which is a dynamic function of mastication, can be affected directly by a decrease in the number of functional teeth and the occlusal force [9,10]. Additionally, chewing movements may be influenced by masticatory muscle activity and perioral muscle strength [11,12]. A recent study even suggested that low gastrocnemius muscle thickness was associated with poor chewing ability among older adults [13]. Physically inactive individuals have been shown to have a greater risk of periodontal disease, which can lead to a decline in chewing ability [14]. Taken together, these findings indicate that factors affecting masticatory performance in older adults include not only oral health-related factors but also physical factors. Currently, there is limited research evaluating the factors affecting masticatory performance in the older population from an oral as well as a systemic health perspective.

Recent studies have suggested that decreased chewing ability in older individuals may be a key factor influencing sarcopenia [15,16,17]. Sarcopenia is a syndrome characterized by progressive and generalized loss of skeletal muscle mass and strength, which is associated with physical disability, poor quality of life, and death [18]. This implies that a decrease in masticatory function causes nutritional imbalance, which in turn affects muscle health. However, this hypothetical triangle of chewing ability–nutrition–sarcopenia also suggests that positive changes in the muscle may lead to improved chewing ability. Interestingly, it has recently been proposed that sarcopenia is a whole-body process that may also affect muscles involved in chewing and swallowing [19]. Murakami et al. [20] found a correlation between the occlusal force and sarcopenia after adjusting for remaining teeth, age, and body mass index (BMI). A decrease in muscle mass can lead to reduced muscle strength, accelerating muscle atrophy and dysfunction, and these changes may affect masticatory ability. In addition, functional muscle decline in older individuals causes constriction of living space, contributing to the loss of mobility or weakness, which negatively affects their ability to move independently and receive dental care. Thus, the importance of comprehensively identifying factors related to masticatory performance from an oral and physical perspective, to maintain healthy aging, has been recognized. This study aimed to evaluate masticatory performance in community-dwelling older adults living in South Korea and to analyze oral and systemic factors that can affect it. The null hypothesis was that there is no correlation between masticatory performance, expressed as mixing ability index (MAI), number of functional tooth units (FTUs), and skeletal muscle mass index (SMI).

## 2. Materials and Methods

### 2.1. Ethical Approval

The study protocol was approved by the institutional review board of our university (IRB No. 1044396-201802-HR-60-01). All procedures of the study complied with the ethical principles for medical research involving human participants, as stipulated in the Declaration of Helsinki (2013 version) by the World Medical Association. This study is reported in accordance with the Strengthening the Reporting of Observational Studies in Epidemiology guidelines [21]. Before the start of the study, the purpose and methods were explained in detail to all participants, and only those who provided informed consent were included. General eligibility was ensured through a questionnaire for screening (see Supplementary Materials, File S1, for the questionnaire).

### 2.2. Participants

The sample size was calculated by applying a linear multiple regression model from G*power 3.1 software (Heinrich-Heine-University Düsseldorf, Düsseldorf, Germany). Based on the results of our preliminary study [22], the number of participants was calculated to be 73, with an effect size (f^2^) of 0.15, an alpha level of 0.05, and a power of 90%. Estimating a dropout rate of about 20%, the total enrollment size was determined to be 88. Healthy adults aged 65 years or older were recruited from among visitors to the community-based Health Promotion Center located in Yeonsu-gu, Incheon, from March to December 2019. Among a total of 88 volunteers, 84 (38 men, 46 women; age 71.40 ± 5.15 years) were selected through an interview to verify their medical and dental history. The study enrolled healthy individuals aged ≥ 65 years without infectious diseases or uncontrolled systemic diseases, who were able to move on their own, and voluntarily expressed their intention to participate. The inclusion criteria were an absence of infectious diseases, age ≥ 65 years, presence of permanent dentition, except for the third molars, and provision of informed consent. The exclusion criteria included factors that could influence the evaluation of masticatory performance, such as uncontrollable systemic diseases, side effects from medications, mental (including cognitive impairment) and physical weakness, nutrient deficiency, hormone therapy, steroid therapy, painful dental caries, a community periodontal index of 4 or higher, orofacial pain, denture wearers, other dental treatment plans, and poor literacy.

### 2.3. General Evaluation

Data on participants’ age, sex, height, weight, BMI, smoking and drinking status, strength training, and systemic disease were collected. Following the author’s instructions, each participant took off their shoes, climbed on the stadiometer, and straightened their neck, waist, and knees for their height and weight measurement. Height was measured with an accuracy of ±0.1 cm; weight was measured with an accuracy of ±0.1 kg. BMI was calculated as the weight (kg) divided by the square of the height (m^2^). Among Asians, a BMI > 25 is classified as obese [23]. Information on smoking, alcohol consumption per week, and continuous strength training for 40 min or more per week within the previous 3 months was recorded. Systemic disease morbidity in the previous 6 months and any ongoing hospital treatment were also recorded.

### 2.4. Skeletal Muscle Mass Index Measurement

Skeletal muscle mass was measured with bioelectrical impedance analysis using an InBody 720 (Bio Space, Seoul, Republic of Korea). After removing accessories attached to the body, including eyeglasses, and wiping bare hands and feet with wet wipes, the participant got on the body composition analyzer, held both handles lightly, and stood still for about 1 min. Appendicular skeletal muscle mass (ASM, kg) was determined from the sum of the upper and lower extremities. The SMI (kg/m^2^) was calculated by dividing the ASM by the square of the height (m^2^). Abnormal SMI values were defined as <7.0 kg/m^2^ for men and <5.7 kg/m^2^ for women, based on the cut-off values specific to Asian older adults [24].

### 2.5. Oral Health-Related Factor Test

An oral examination was performed in a dental unit chair by a trained dental hygienist. The number of FTUs was defined as the number of pairs of opposing teeth in the upper and lower jaws, excluding third molars [25]. The number of FTUs was scored as 2 FTUs for molars and 1 FTU for premolars. A person with complete dentition, excluding third molars, would thus have a total of 12 FTUs [9]. Stimulated salivary flow rate (mL/min) was measured by asking the participants to chew paraffin wax for 5 min and collecting the irritant saliva secreted in a 50-mL tube.

### 2.6. Assessment of Masticatory Performance

**Figure 1:**
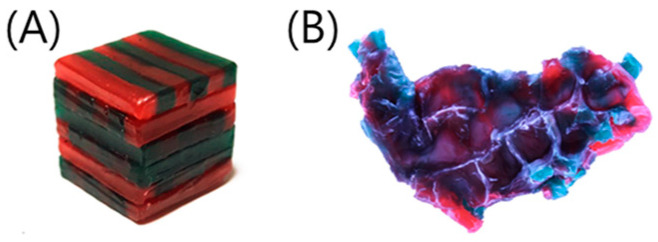
Preparation of a two-color wax cube (**A**) and a chewed wax cube (**B**) [26].

Masticatory performance was assessed using the MAI, which is calculated by comprehensively evaluating the total area, degree of perforation, and degree of color mixing after masticating a wax cube. The wax cube, composed of red and green utility wax rods arranged as 12 mm × 12 mm × 12 mm (Daedong Industrial Co., Ltd., Daegu, Republic of Korea) without overlap [26], was masticated 10 times (Figure 1). One cube was used for each side of the mouth. Subsequently, both the front and back sides of the masticated specimen were photographed using a digital camera (Canon EOS 500D, Canon Korea Consumer Imaging Inc., Seoul, Republic of Korea), while maintaining the same distance between the light source, the lens, and the specimen. The camera was set to ISO 100, a shutter speed of 1/40, and an aperture of 5.6. The images were analyzed using a specialized program (Image-pro 10.0, Media Cybernetics Inc., Silver Spring, MD, USA). Total projection area (TPA) in mm^2^, projection area less than 50 µm in thickness (P) in mm^2^, maximum length (ML) in mm, maximum breadth (MB) in mm, red area (RA), and green area (GA) were first extracted. These values were subsequently used to calculate the ratio of the area mixed with two colors (MIX), calculated as 100 − (RA + GA)/P × 100; the ratio of the area below 50 μm in thickness to TPA (TR), calculated as 100 − P/TPA × 100; the proportion of maximum length to breadth (LB), calculated as ML/MB; and the shape factor (FF), which shows how flat the sample is (ML^2^ × π/4 × TPA × 100). These values were finally used to calculate the MAI [26,27], in which the average values of the front and back sides of the masticated wax cube for each participant were recorded as the average MAI of two wax cubes masticated to the right and left, respectively [11]. The MAI was normalized to 0–100 points, where a higher MAI score indicated a higher masticatory ability.

### 2.7. Statistical Analyses

All data were analyzed using SPSS statistics version 26.0 (IBM, Chicago, IL, USA). The significance level was set to *p* < 0.05. The Shapiro–Wilk test was used to determine the normality of the data. Descriptive statistics with continuous normal distribution are presented as mean ± standard deviation (SD), and non-normally distributed variables are presented as the median and interquartile range (IQR; 25th and 75th percentiles). Categorical variables are expressed as numbers and percentages. Continuous variables were compared using the Mann–Whitney *U* test, Kruskal–Wallis test, or independent *t*-test for MAI group differences. Categorical variables were compared using the chi-square test. Correlation coefficients between the MAI and other variables were calculated using Spearman’s rank correlation coefficient. Linear multiple regression analyses were carried out to test each predictor variable’s relationship with the MAI, after controlling for the other factors. All the predictor variables were entered into the model using the ‘enter’ method.

## 3. Results

Table 1 shows the sociodemographic characteristics, oral and general health-related factors, and muscle-related indicators separated by sex. The mean age of the participants was 71.40 ± 5.15 years (men, 72.95 ± 5.62; women, 70.13 ± 4.40). The mean SMI was significantly higher in men than in women by 1.25 times (*p* < 0.001). Among the participants, 18 (21.4%) had an SMI lower than the diagnostic criteria for sarcopenia, of which 14 (77.8%) were women. The median number of FTUs was 10 (9.00–10.00) and did not differ significantly by sex. The median MAI of all participants was 68.08 (51.78–75.87), and the MAI of men was significantly higher than that of women, by 1.17 times (*p* = 0.023).

**Figure 2:**
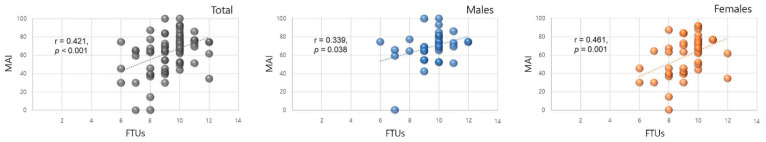
Correlation between functional tooth units (FTUs) and the mixing ability index (MAI).

**Figure 3:**
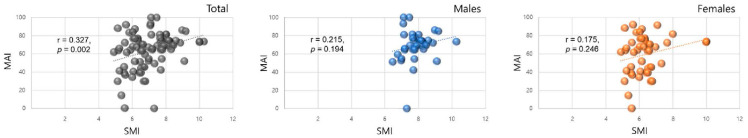
Correlation between the skeletal muscle mass index (SMI) and the mixing ability index (MAI).

**Figure 4:**
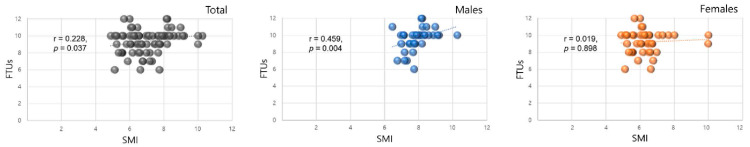
Correlation between the skeletal mass index (SMI) and functional tooth units (FTUs).

Only sex was significantly related to the MAI among the sociodemographic, lifestyle, and systemic disease-related variables (Table 2). As seen in Table 3, FTUs and the SMI showed a moderate correlation with the MAI (r = 0.421, *p* < 0.001 and r = 0.327, *p* = 0.002, respectively). Analyzing variables by sex revealed that the MAI increased significantly with FTUs in both men and women (r = 0.339, *p* = 0.038 and r = 0.461, *p* = 0.001, respectively, Figure 2). No significant correlation was found between the SMI and the MAI in either group (Figure 3). However, a moderate correlation was found between the SMI and FTUs among men, as shown in Figure 4 (r = 0.459, *p* = 0.004).

When testing the effect of the various factors on the MAI (Table 4), we found that an increase in FTUs and the SMI significantly increased the MAI (B = 5.388, *p* = 0.001 and B = 4.016, *p* = 0.017, respectively, adjusted R^2^ = 0.203). However, in our sex-adjusted model (Table 4), the MAI increased only with FTUs (B = 5.405, *p* = 0.001, adjusted R^2^ = 0.198). Additionally, when the analysis was split by sex (Table 5), the MAI showed a tendency to increase by about 4.4 and 6.9 times for each FTU unit in men and women (B = 4.442, *p* = 0.037, R^2^ = 0.116 and B = 6.898, *p* = 0.003, R^2^ = 0.183, respectively). However, in Model 2, to which SMI was added, there was a difference in the results between men and women. The increase in FTUs or the SMI did not significantly affect the increase in the MAI among men (B = 4.083, *p* = 0.075 and B = 1.686, *p* = 0.663, respectively, adjusted R^2^ = 0.070), while among women, the MAI increased significantly by about 6.7 times for every FTU unit increase, even after adding the SMI (B = 6.685, *p* = 0.004). In addition, as the SMI increased by one unit, the MAI showed a tendency to increase by about 4.0 times; however, this result was not statistically significant (B = 4.005, *p* = 0.151, adjusted R^2^ = 0.186).

## 4. Discussion

This study assessed the factors affecting the masticatory performance of functionally independent older adults from an oral and physical perspective. We found that the MAI was associated with sex, the SMI, and the number of FTUs (Table 2 and Table 3). When testing whether these factors could predict a change in the MAI, we found that increases in the number of FTUs and the SMI significantly increased the MAI. However, after adjusting for sex, only the number of FTUs had a significant effect (Table 4). This can be explained by FTUs and the MAI having a stronger correlation than that between the SMI and MAI (Table 3). Our result is consistent with that of a previous study reporting that the number of FTUs was a key factor influencing the MAI [28]. Further, SMI was strongly influenced by sex; our findings showed that the SMI was approximately 1.25 times higher in men than in women (Table 1). Therefore, the correlation between the SMI and the MAI that appeared in the entire group disappeared when adjusting for sex (Figure 3), suggesting that the influence on the MAI can vary depending on sex.

When the regression analysis was split by sex, the association between the MAI and the number of FTUs in men was no longer significant after the SMI was added. However, in women, the number of FTUs was independently associated with the MAI after adjusting for the SMI (Table 5). This discrepancy could be due to the oral and physical characteristics of the participants, who were functionally independent healthy elderly people with well-managed oral health. Nevertheless, the sex of the participants caused physical differences, such as those in height, weight, and skeletal muscle mass, resulting in the SMI being approximately 1.25 times higher in men than in women (Table 1). Commonly, an association between chewing difficulties and aging is well documented; decreased masticatory functions associated with age are related to functional tooth loss and decreased occlusal force [29]. However, our findings suggested that the number of FTUs does have a decisive effect on the MAI in men. This disagreement is presumably caused by the correlation between the MAI, SMI, and FTUs. The SMI showed a significant correlation with the number of FTUs in men (Figure 4), which might have eliminated the effect of the FTUs on the MAI in Model 2 of men (Table 5). The correlation between the FTUs and SMI could be supported by a previous study reporting that oral frailty could be a risk factor for physical frailty, mortality, and sarcopenia [30]. Therefore, our findings suggest that the number of FTUs may be not a major factor in older adults if the number of functional teeth and the amount of skeletal muscle mass are maintained above a certain cut-off limit. That is, the number of FTUs by itself may not be a risk factor for masticatory dysfunction in older adults with well-maintained oral and general health.

In contrast, among women, there was a significant increase in the MAI as FTUs increased, even when the SMI was added (Table 5). This may be because the SMI had no significant correlation with either the MAI or FTUs in women, given their lower SMI compared to that of men. Our findings are in disagreement with the results of a previous study in which low handgrip strength was significantly associated with chewing ability after adjusting for all confounders in women and an association was observed between the number of functional teeth and chewing ability in men [13]. The reasons could be attributed to the following. The participants of this previous study had significant differences in the SMI according to sex, and the SMI of only elderly women was in the normal range. In addition, handgrip strength may be a more sensitive indicator of chewing ability than SMI. Nevertheless, our findings suggest that older adults with less skeletal muscle mass may have no choice but to rely on FTUs to improve their masticatory performance.

However, despite the SMI having no significant effect on the MAI in both men and women, we cautiously argue that the possibility of the SMI affecting the MAI cannot be completely excluded; in this regard, the lack of statistical significance may have been due to the small sample size. Our approach is supported by articles reporting *p* values and yet renouncing dichotomous statements (significant/non-significant) [31,32]. Therefore, we focused on interpreting the implications of the data trend shown among the older population. A recent study showed that chewing ability had negative associations with some muscle-related parameters, such as handgrip strength and gastrocnemius muscle thickness [13]. These findings could support our observation that muscle-related factors affect masticatory performance. Furthermore, it has been reported that the decrease in masseter muscle thickness can negatively affect masticatory performance [22]. According to another study, the masseter muscle thickness may decrease as the appendicular skeletal muscle mass decreases [33]. To the best of our knowledge, few studies have explained the relationship between the SMI and chewing ability. Therefore, future studies need to prove the effect of muscle-related factors on the MAI with a reliable study model.

The limitations of this study include a small participant sample size, which may have prevented appropriate comparison between the male and female participants. This resulted in a low effect size of the FTUs or SMI on the MAI. Future studies should include a larger sample. The limitations of the cross-sectional study format prevented establishment of a causal relationship between the MAI and the number of FTUs. To investigate a causal relationship between the MAI and oral and body factors, a longitudinal study design should be considered in the future. This study evaluated only muscle mass, not muscle strength or physical performance; additionally, the properties of masticatory muscles were not examined. Body-related factors that affect masticatory performance, such as those related to sarcopenia and masticatory muscles, should be considered in future studies. Finally, this study included older individuals who participated in a community health promotion center program. Thus, they were functionally independent, older urban adults who were highly interested in their health and took good care of it. Therefore, the results from this study may not translate to less independent older adults.

Despite the limitations of this study, our results suggest that oral and physical-related factors influence the MAI in older adults, although the MAI can be affected by sex. The findings serve as a foundation for future research and to understand and the masticatory performance–nutrition–physical frailty triangle in further detail.

## 5. Conclusions

The number of FTUs had a significant effect on the MAI among women with low muscle mass, whereas there was no significant effect of the SMI on the MAI; however, its influence should not be dismissed. A multidisciplinary approach, including oral health care and personalized physical training, may be more effective in managing the health of older adults and could significantly contribute to preserving muscle function as well as oral health in the older population.

## Figures and Tables

**Table 1:** Characteristics of the participants.

Variables	Total	Men	Women	*p*-Values ^†^
	(N = 84)	(N = 38)	(N = 46)	
Age (years)	71.40 ± 5.15	72.95 ± 5.62	70.13 ± 4.40	0.014
65–69	38 (45.2)	14 (36.8)	24 (52.2)	0.247
70–74	22 (26.2)	9 (23.7)	13 (28.3)	
75–79	15 (17.9)	9 (23.7)	6 (13.0)	
80–85	9 (10.7)	6 (15.8)	3 (6.5)	
Height (cm)	161.65 ± 8.81	169.29 ± 4.98	155.35 ± 5.75	<0.001
Weight (kg)	63.78 ± 10.34	71.05 ± 8.67	57.77 ± 7.34	<0.001
Body mass index (kg/m^2^)	24.34 ± 2.88	24.79 ± 2.80	23.96 ± 2.92	0.194
Normal	47 (56.0)	19 (50.0)	28 (60.9)	0.437
Obesity	37 (44.0)	19 (50.0)	18 (39.1)	
Smoking				
Yes	4 (4.8)	4 (10.5)	0 (0.0)	0.082
No	80 (95.2)	34 (89.5)	46 (100.0)	
Number of drinks (per week)				
0	61 (72.6)	18 (47.4)	43 (93.5)	<0.001
1–2	16 (19.0)	13 (34.2)	3 (6.5)	
3–4	7 (8.3)	7 (18.4)	0 (0.0)	
Strength training				
Yes	6 (7.1)	4 (10.5)	2 (4.3)	0.504
No	78 (92.9)	34 (89.5)	44 (95.7)	
Hypertension				
Yes	32 (38.1)	14 (36.8)	18 (39.1)	1.000
No	52 (61.9)	24 (63.2)	28 (60.9)	
Diabetes				
Yes	18 (21.4)	7 (18.4)	11 (23.9)	0.731
No	66 (78.6)	31 (81.6)	31 (76.1)	
Dyslipidemia				
Yes	7 (8.3)	1 (2.6)	6 (13.0)	0.186
No	77 (91.7)	37 (97.4)	40 (87.0)	
Osteoporosis				
Yes	3 (3.6)	0 (0.0)	3 (6.5)	0.311
No	81 (96.4)	38 (100.0)	43 (93.5)	
Appendicular skeletal muscle mass (kg)	18.59 ± 4.54	22.59 ± 2.39	15.28 ± 2.94	<0.001
Skeletal muscle mass index (kg/m^2^)	7.02 ± 1.21	7.88 ± 0.76	6.31 ± 1.04	<0.001
Normal	66 (78.6)	34 (89.5)	32 (69.6)	0.052
Abnormal	18 (21.4)	4 (10.5)	14 (30.4)	
Stimulated saliva secretion rate (mL/min)	2.00 (1.40–2.40)	2.20 (2.00–3.00)	1.60 (1.00–2.00)	<0.001
Functional tooth units	10.00 (9.00–10.00)	10.00 (9.00–10.00)	9.50 (8.00–10.00)	0.174
Mixing ability index	68.08 (51.78–75.87)	70.72 (64.51–77.15)	63.93 (41.30–74.18)	0.023

All values are presented as number (%), median (25–75%), or mean ± standard deviation. ^†^ *p*-values obtained from the chi-square test, independent *t*-test, and Mann–Whitney *U* test at α = 0.05.

**Table 2:** Mixing ability index-related variables.

Variables	N	Mixing Ability Index	*p*-Values ^†^
Sex			
Male	38	70.72 (64.51–77.15)	0.023
Female	46	63.93 (41.30–74.18)	
Age (years)			
65–69	38	65.52 (51.24–74.51)	0.865
70–74	22	68.38 (59.10–81.80)	
75–79	15	70.35 (47.86–75.89)	
80–85	9	73.42 (65.53–74.08)	
Body mass index (kg/m^2^)			
Normal	47	67.75 (53.44–75.52)	0.838
Obesity	37	68.41 (49.29–74.68)	
Smoking			
Yes	4	69.36 (68.89–84.62)	0.322
No	80	67.43 (51.47–75.47)	
Number of drinks (per week)			
0	61	67.38 (45.59–74.68)	0.269
1–2	16	72.60 (64.78–76.43)	
3–4	7	69.36 (66.61–77.67)	
Exercise			
Yes	6	74.08 (29.88–74.68)	0.976
No	78	67.62 (52.00–76.26)	
Hypertension			
Yes	32	69.86 (53.21–75.47)	0.951
No	52	67.43 (51.47–75.52)	
Diabetes			
Yes	18	63.42 (40.61–72.07)	0.059
No	66	69.36 (54.41–76.53)	
Dyslipidemia			
Yes	7	49.29 (45.30–64.61)	0.156
No	77	69.00 (54.41–76.26)	
Osteoporosis			
Yes	3	61.57 (56.64–72.48)	0.910
No	81	68.41 (52.00–74.68)	

All values are presented as median (25%–75%). ^†^ *p*-values obtained from the Mann–Whitney *U* test or Kruskal–Wallis test at α = 0.05.

**Table 3:** Correlations between mixing ability index and each variable (N = 84).

Variables	r	*p*-Values ^†^
Functional tooth units	0.421	<0.001
Skeletal muscle mass index (kg/m^2^)	0.327	0.002
Stimulated saliva secretion rate (mL/min)	0.108	0.327
Body mass index (kg/m^2^)	0.037	0.739

^†^ *p*-values obtained from Spearman correlation analysis at α = 0.05.

**Table 4:** Factors associated with mixing ability index.

Predictor Variables	Model 1			Model 2				Model 3			
	Unstandardized B	95% CI for B	*p*-Values	Unstandardized B	Standardized β	95% CI for B	*p*-Values	Unstandardized B	Standardized β	95% CI for B	*p*-Values
FTUs	6.189	3.142–9.237	<0.001	5.388	0.355	2.355–8.421	0.001	5.405	0.356	2.360–8.450	0.001
SMI				4.016	0.244	0.728–7.304	0.017	3.123	0.190	−1.189–7.436	0.153
Sex								3.308	0.083	−6.977–13.593	0.524

Enter multiple regression analysis was performed with α = 0.05. CI, confidence interval; FTUs, functional tooth units; SMI, skeletal muscle mass index.

**Table 5:** Factors associated with mixing ability index by dividing into subgroups according to sex.

Predictor Variables	Men							Women						
	Model 1			Model 2				Model 1			Model 2			
	Unstandardized B	95% CI for B	*p*-Values	Unstandardized B	Standardized *β*	95% CI for B	*p*-Values	Unstandardized B	95% CI for B	*p*-Values	Unstandardized B	Standardized *β*	95% CI for B	*p*-Values
FTUs	4.442	0.290–8.594	0.037	4.083	0.313	−0.436–8.601	0.075	6.898	2.473–11.324	0.003	6.685	0.415	2.303–11.067	0.004
SMI				1.686	0.075	−6.098–9.470	0.663				4.005	0.197	−1.514–9.525	0.151

Enter multiple regression analysis was performed with α = 0.05. CI, confidence interval; FTUs, functional tooth units; SMI, skeletal muscle mass index.

## Data Availability

The data presented in this study are available from the corresponding author upon request.

## Supplementary Materials

The following supporting information can be downloaded at: https://www.mdpi.com/article/10.3390/ijerph192315742/s1, File S1: Questionnaire for screening for general eligibility.
