# Supplementary material for: Factors Affecting Masticatory Performance of Older Adults Are Sex-Dependent: A Cross-Sectional Study
Source: Int J Environ Res Public Health. 2022 Nov 26;19(23):15742. doi: 10.3390/ijerph192315742 (PMC9735781; doi:10.3390/ijerph192315742)
Supplement: Supplementary file 1 [file ijerph-19-15742-s001.zip › ijerph-2055934-supplementary.pdf]

File S1: Questionnaire for screening for general eligibility

Q.1. Are you suffering from infectious diseases such as hepatitis and AIDS?

Q.2. Are you currently suffering from diseases such as high blood pressure, diabetes, hyperlipidemia, and osteoporosis? If Yes, are you taking medicine?

Q.3. Can you move on your own?

Q.4. Do you have any mental problems such as cognitive impairment?

Q.5. Are you eating normally?

Q.6. Do you take any medicine regularly for hormone therapy or steroid therapy?

Q.7. Do you have any painful teeth?

Q.8. Do you have gum disease?

Q.9. Are you undergoing dental treatment?

Q.10. Are you wearing dentures?

Q.11. Can you read the newspaper?
